# Study on Market Stability and Price Limit of Chinese Stock Index Futures Market: An Agent-Based Modeling Perspective

**DOI:** 10.1371/journal.pone.0141605

**Published:** 2015-11-16

**Authors:** Xiong Xiong, Ding Nan, Yang Yang, Zhang Yongjie

**Affiliations:** 1 College of Management and Economics, Tianjin University, Tianjin, 300072, PR China; 2 China Center for Social Computing and Analytics, Tianjin University, Tianjin, 300072, PR China; East China University of Science and Technology, CHINA

## Abstract

This paper explores a method of managing the risk of the stock index futures market and the cross-market through analyzing the effectiveness of price limits on the Chinese Stock Index 300 futures market. We adopt a cross-market artificial financial market (include the stock market and the stock index futures market) as a platform on which to simulate the operation of the CSI 300 futures market by changing the settings of price limits. After comparing the market stability under different price limits by appropriate liquidity and volatility indicators, we find that enhancing price limits or removing price limits both play a negative impact on market stability. In contrast, a positive impact exists on market stability if the existing price limit is maintained (increase of limit by10%, down by 10%) or it is broadened to a proper extent. Our study provides reasonable advice for a price limit setting and risk management for CSI 300 futures.

## Introduction

On April 16, 2010, CSI 300 futures succeeded in listing on the China Financial Futures Exchange. The structure of China’s domestic capital market has improved through the formation of a spot-future cross-market structure. The stock index futures is an ideal, systemic risk-hedging tool for financial market investors. However, due to its leverage properties and the hedging strategies, it may lead to market shocks and increase the systematic risk (such as Dow Jones Industrial Average’s intraday flash crash caused by the E-mini future contracts.) Thus, effectively managing the risk of the stock index futures market and cross-market to prevent excessive volatility and stabilize the market is critically important for financial market stability. Price limits, which stabilize the stock index futures market through limiting the maximum trading price fluctuations within one trading day, have been used most commonly in many countries including China.

Following the worldwide crashes in 1987 and 1989, research has started to focus on financial market stability mechanisms including price limits. However, viewpoints are mixed on price limits’ function of risk management and market stabilization. Advocates argue that price limits can curb market overreaction and reduce market volatility by providing a cooling-off period for investors to reevaluate information. Papers [1] and [2] conducted empirical studies on American treasury futures markets and found that price limits is useful for reducing the market volatility. Paper [3] analyzed China's stock market by comparing a period with price limits (1997–2000) to a period without price limits (1992–1996) and found price limits could facilitate price discovery, moderate transitory volatility, and mitigate abnormal trading activity, even for poorly performing stocks with tighter price limits.

Other critics argue that price limits have several adverse effects for market stability. First, price limits have volatility spillover effects because they can generate higher volatility on subsequent trading days. This argument is supported by an empirical study [4] that researched stocks listed on the Tokyo Stock Exchange. The authors found that the volatility of stock prices do not return to normal level after the stocks reached price limits. Paper [5] researched the call options listed on the Warsaw Stock Exchange and examined the existence of volatility spillover effect. The second adverse effect is price discovery delaying. Paper [6] studied the American stock market and found that trading halts can delay the price reaching an equilibrium level. Studies [7] and [8] support this hypothesis. Third, some studies found evidence that price limits can interfere with trading because the liquidity of limit-hit financial instruments is decreased such as in [9], [10], and [7]. In addition, paper [6] mentioned that the price would accelerate toward the limits level when it gets closer to the limits, which is a phenomenon known as the magnet effect of price limits. Paper [11] proved the existence of this effect by empirical studying the Taiwan stock market. Paper [12] argued that behavioral investors who believe in the price trends could act in a way that produces a magnet effect.

Some studies have attempted to find optimal price limits for researched markets by building optimization models [13–15]. In addition, paper [16] adopted an artificial stock market to analyze price limits in China and found that reasonably enlarged existing price limits could moderate the volatility of stock returns without considering some factitious factors.

These studies show important research findings on the effectiveness of price limits. We also find that some of the previous findings are controversial based on same market or the same financial instrument, as in papers [4] and [17]. These studies indicate two problems: the first one, whether the price limit mechanism is helpful for market stability and depends on the specific financial market’s condition; the second problem is that analyzing methods of previous studies have limitations. Empirical methods restrict studies to obtain data based on existing markets, which means they cannot eliminate the impact of existing price limits. In addition, complex mathematical methods are difficult to demonstrate changes in the real market since the models are built based on a range of assumptions and the ability to obtain data is still limited. Moreover, even if some studies use artificial financial-modeling methods, the market structure is single not cross. And the single market mechanism is hard to stimulate the cross-market transactions of different kinds of investors. In China, although the CSI 300 futures were launched four years ago, there are few researches exist on the effectiveness of price limits for market stability. These studies show important research findings of the function of price limits.

To overcome the limitations of previous studies, this paper adopts an agent-based computational modeling method, which provides a new perspective in analyzing complex cross-market transactions. Previous studies have created some cross-market artificial financial markets. SumWEB is a cross-market platform in which stock market and futures market co-exist and provides a basic platform for analyzing cross- market arbitrage and price manipulation [18]. U-mart is another popular basic simulation platform to simulate cross-market transactions by using the Japan stock index J30 as the underlying asset [19]. In this paper, we adopt an agent-based computational model set based on a previous study by our academic research team [20]. It is a spot-future cross-market structure that coincides with the main characteristics of the Chinese stock market and the CSI 300 futures market. Compared to the single market, this cross one is more suitable to conduct research in stock index future market. To be specific, we stimulate the market operations under different price limits settings through modifying parameters on the platform and then use appropriate liquidity indicators and volatility indicators to analyze the results. Our simulation results provide reasonable advice for the price-limit settings of CSI 300 futures.

The rest of the paper is organized as follows. Section 2 provides a brief description of the model. Section 3 introduces the liquidity and volatility indicators in our experiments. Section 4 provides an analysis of the experimental results. Finally, section 5 concludes the paper.

## The Model

In this artificial financial market, the stock market and the stock index futures market co-exist. There are several kinds of stocks that could be traded in stock market but only one kind of stock index future that can be traded in the stock index futures market. The investors can be divided into three types in each market and include informed traders, uninformed traders, and noise traders. In addition, a type of spot-future arbitrager exists who trades between the two markets. For each type of trader, the investment demands are endogenously determined and subject to constraints from wealth, risk management level, and trading mechanisms in the markets. This almost coincides with the existing trading mechanisms of the Chinese stock market and the CSI 300 futures market.

### 2.1 Assets

There are five stocks and one stock index future in the artificial market. The underlying asset of the stock index future is the stock index that is constructed based on these five stocks. The common value of each stocks common value’s calculation and the parameter settings draw on paper [20].

#### 2.1.1 The common value of stock

The stock's common value is given by

$${\text{v}}_{\text{i},\text{t}+1}^{*}=\left(1+\emptyset_{\text{i}}+\sigma_{\text{i},\varepsilon }\varepsilon_{\text{t}+1}\right){\text{v}}_{\text{i},\text{t}}^{*}.$$ (1)

Here, ∅_i_ represents the dividend growth rate of stock i, namely the randomwalk drift of the common value. The time interval t in the simulation corresponds to 5 seconds in the real world. Since the time interval is extremely short, we set the growth rate to be zero, namely ∅_i_ = 0. We assume ε_t_ ∈ N(0,1).σ_t,ε_ > 0 represents the standard deviation in this diffusion process. Parameters of the five stocks are set as shown in Table 1.

**Table 1:** The Parameters of the Five Stocks.

Stock number	Initial value	S.D. of disturbance	Stock's share(100 million)
**1**	10	0.0007	50
**2**	20	0.0007	40
**3**	30	0.0003	60
**4**	40	0.0003	30
**5**	50	0.0005	50

#### 2.1.2 Stock index

The unit of the stock index is called "point," and the base period value is 3,000 points. The stock index is calculated by the weighted composite price index method:

$${\text{I}}_{\text{t}}=\frac{{\text{M}}_{\text{t}}}{{\text{M}}_{0}}\times 1000=\frac{\left(\sum {\text{P}}_{\text{i},\text{t}}{\text{S}}_{\text{i},\text{t}}\right)}{{\text{M}}_{0}}\times 1000.$$ (2)

Here, M_t_ stands for the market value of stock index at t period, which is sum of the current stock price P_i,t_ multiplied by the number of share outstanding S_i,t_. M_0_ is that of the base period.

#### 2.1.3 The common value of the index future

The stock index future’s common value is calculated according to the future's theoretical value:

$${\text{v}}_{\text{F},\text{t}}={\text{I}}_{\text{t}}{\left(1+\text{r}\right)}^{\text{T}-\text{d}+1}.$$ (3)

Here, I_t_ is the current index, *T* is the expiration date, and d is the listing date of this stock index future.

### 2.2 Markets

There are two artificial financial markets, a stock market and a futures market. Considering the need for high-frequency trading, both the markets adopt a continuous double auction trading mechanism and *T* + 0 rule. Traders could place both limited orders and market orders. Buying limited orders whose prices are equal to or higher than the best ask price and selling limit orders whose prices are equal to or lower than best bid price are treated as market orders and executed instantly. According to the rules on the China financial futures exchange, limit orders have a certain life span, and unexecuted limit orders will be cleared at the end of the day.

In the simulation, the time interval t corresponds to 5 seconds in the real world, and each time interval may contain several transactions or no transactions.

Short selling is not available in the stock market but is available in the futures market. Thus, the only available arbitrage strategy for cross-market arbitrageurs is the positive side, namely buying stocks and selling futures.

The stock index future market requires margins and settles the balance of each margin account after the market closes every day. Once an investor’s equity is less than the minimum margin requirement for holding positions, his positions will be forced to close one by one the next day, until his equity is not less than the required minimum margin.

The transaction price of the market P_i,t_ is the average price of the several transactions taking place during *t*, and if no transaction is performed the price is equal to that of the last time, namely P_i,t_ = P_i,t−1_.

No transaction costs exist for either stocks or futures.

If the wealth of an investor is smaller than a certain amount, he will be identified as bankrupt and exit the market. Another investor who is identical to the former one in investor type, initial wealth, and stocks or futures investments will enter the market.

### 2.3 Investors’ Structure and Behaviors

Seven kinds of investors exist in the markets. Three of these kinds only trade stocks, namely informed traders, uninformed traders, and noise traders. They are designed to randomly choose one stock and invest in it permanently. Similarly, there are also three types of investors that trade only in the futures market. The spot-future arbitrageurs trade in both the stock market and the futures market. The spot-futures arbitragers watch the relationship between the stock index and the price of the stock index futures in real time. Once the futures price is higher than the upper boundary of the no-arbitrage range and reaches the arbitrageur’s expected profit point, he will take positions, buy stock portfolios, and sell futures at the same time. He will close the positions (sell stock portfolios and buy futures) once the futures price falls back to the arbitrage caps, otherwise, he will hold the positions to due. The arbitrageurs seek the immediate execution of their orders and thus only place market orders. To realize risk-free arbitrage, the spot-futures arbitrageurs allocate their wealth between stock portfolios and futures and maintain the ratio of the margin for all of the assets within a specific range, thus keeping the account safe.

#### 2.3.1 Traders’ expectations on assets price

Since the expectations of the three types of investors who trade only in the stock market are similar to those who trade only in the futures market, for the sake of simplicity, we talk about them together and remove the subscript that stands for the asset number. Assume that all investors know the current common value of a stock, while there is divergence in the futures common value.

*i*. *Informed trader*: Informed trader i clearly know the stock's common value in the following periods τ when he enters the markets, and the expected price is ${\hat{P}}_{t+\tau }^{i}=v_{t+\tau }$. However, as for the informed traders in the futures market, they cannot accurately know V*t+τ* as they are not able to predict I_t+τ_ accurately. Here we assume that they learn ${\hat{I}}_{t+\tau }$ through the stock’s common value, thus the expected price ${\hat{p}}_{t+\tau }^{i}$ will be

$${\hat{p}}_{t+\tau }^{i}={\hat{I}}_{t+\tau }{\left(1+r\right)}^{T-d+1}=\frac{\left(\sum v_{i,t+\tau }S_{i,t}\right)}{M_{0}\times {\left(1+r\right)}^{T-d+1}}\times 3000.$$ (4)

*ii*. *Uninformed trader*: Uninformed traders cannot know the future common value v_t+τ_ but they know the current common value v_t_. They obtain the expected prices by mixing three sources, including the current common value v_t_, τ period average transaction prices $\bar{P_{\tau }}$, and current mid-point of bid and ask prices P_m_. The expected price is given by

$${\hat{p}}_{t+\tau }^{i}=\frac{\left(a^{i}v_{t}+b^{i}{\bar{p}}_{\tau }+c^{i}p_{m}\right)}{\left(a^{i}+b^{i}+c^{i}\right)}.$$ (5)

*iii Noise trader*: The expected price of noise trader i is randomly chosen within the five levels from the bid and ask prices in the order book, which are given by

$${\hat{p}}_{t+\tau }^{i}={\mathrm{bid}}_{5}+{\mathrm{rand}}_{t}^{i}\times ({\mathrm{ask}}_{5}-{\mathrm{bid}}_{5}).$$ (6)

Here, bid_5_ is the fifth low quoted price in order list, and bid_5_ is the fifth high quoted price in order list.

#### 2.3.2 The proportion of different kinds of investors

Paper [21] empirically analyzes the proportion of informed traders as between 11.21% and 18.62% on the Shanghai Stock Exchange. Paper [22] finds the average proportion of noise traders on China’s stock market is 58.14%. According to this research, we set the proportion of informed traders, uninformed traders, and noise traders as 12%, 30% and 58%, respectively, in this simulation.

Paper [23] calculated the investors’ trading frequency in a trading day through the high frequency data provided by the Chinese Financial Futures Exchange. The authors then divided the investors' type in four types based on the different levels of trading frequency, namely informed trader, uninformed trader, noise trader, and cross market arbitrager, and the proportions at 3.33%, 80.18%, 10.05%, and 6.44%. Based on the results of [23], in this simulation we set the proportions of informed trader, uninformed trader, noise trader and cross-market arbitrager at 4%, 79%, 10% and 7% respectively.

#### 2.3.3 Order size

Given price *p*, an investor’s optimal positions depend on his utility function. Following the demands determined in paper [24], we assume that investors are absolutely risk averse, namely, that they make their investment decisions by maximizing the CARA utility function and their optimal position is

$$\pi^{i}(p)\frac{\ln\left({\hat{p}}_{t+\tau }^{i}/p\right)}{\alpha^{i}V_{t}^{i}p},$$ (7)

where α^i^ is the absolute risk averse coefficient of investor*i*, $V_{t}^{i}$ is the variance of expected return of investor i, ${\hat{p}}_{t+\tau }^{i}$ is expected price at period t+r^i^, which is different for different types of investors, and *p* is the order submission price. If the demand quantity π^i^(p) is larger (smaller) than the investor's current position, then the investor buys (sells). $V_{t}^{i}$ is estimated by the variance of past returns:

$${V_{t}^{i}=\frac{\sum_{j=1}^{\tau }\left(r_{t-j}-{\overset{-}{r}}_{t}^{i}\right)}{\tau^{i}}}^{2},$$

$${\overset{-}{r}}_{t}^{i}=\frac{\sum_{j=1}^{\tau }r_{t-j}}{\tau^{i}}=\frac{\sum_{j-1}^{\tau }\;\ln\left(p_{t-j}/p_{t-j-1}\right)}{\tau^{i}}.$$ (8)

### 2.4 Parameter Settings

Based on different economic environments and the degree of market development, price stabilization measures vary within countries. In order to explore the suitable price limits in the CSI 300, we simulate five experiments with different price limits and the price-limit settings are shown in Table 2. In experiment 2, the price limit is equal to the real level, that is, the limit up is by 10% and the limit down is by 10%. In experiment 1, we draw on the experience of the Japan NIKKEI 225, which uses a relatively strict price limit of 6% for both the top limit and the bottom limit. In experiment 3, we keep the top-limit level at 10% but broaden the low-limit level to 20%. In experiment 4, we remove the top limit and broaden the low-limit level to 15%. According to the British FISE 100, in experiment 5, we do not impose a price limit, namely NPL (no price limit). To eliminate the randomness, each experiment is run five times to observe the general rule.

**Table 2:** Price-Limit Settings.

Experiment	1	2	3	4	5
**Level**	6%, 6%	10%, 10%	10%, 20%	NPL, 15%	NPL, NPL
**Country**	Japan NIKKEI 225	CSI 300			British FISE 100

Each experiment runs for 60522 periods, which represent 21 days, namely 2882 periods each day. In the model, most of these parameters are consistent with the market structure and trading rules on the Shanghai Stock Exchange and the China Financial Futures Exchange. The risk-free rate in the market is based on the 21-days interbank pledged repo rate of 4.26%. The order book is cleared every day in both the stock market and the futures market. The minimum order size required in the stock market is 100 shares. The tick size is 0.01 Chinese *yuan* for stocks and 0.2 Chinese *yuan* for futures. The minimum margin rate is 18%, which is the margin rate required by brokers in real trading situations. The multiplier of futures contract in our model is 300 Chinese *yuan* per point, which is also consistent with the IF1 009 contract.

For the three types of investors in the stock market, each investor permanently invests in one stock, which is randomly selected before the program begins. The initial positions are allocated randomly in the range [300, 1500], and the initial cash is equal to the initial values of all the investor’s stocks. For the three types of investors in the futures market, each investor’s initial wealth is 3 million, and for each spot-futures arbitrageur, the amount is 10 million. The order-submission interval for the six kinds of single-market investors obeys an exponential distribution, that is, the number of orders during one period follows a Poisson distribution. At the same time, for these six types of single-market investors, the order-submission interval is equal to the order life. If the investors find limited orders submitted before are unexecuted or not fully executed when they reenter the market, they will cancel the former orders first and then place new orders. For uninformed traders, the variables a^i^, b^i^, and c^i^, which represent the weights when forecasting prices, are randomly chosen from [0, 1]. The capital safety ratio for futures investors, which is the wealth share for futures investment, is no more than 60%. As for arbitrageurs’ ex-ante expected profits for every futures contract—which should be enough to compensate the execution costs when trading in both two markets—we set them randomly in the range [10, 20], which are equivalent to [50, 100] index futures’ tick sizes. Each investor’s wealth will be updated at the end of every period. If one investor’s wealth is less than the capital to buy 100 shares of his invested stock, he goes bankrupt and exits the market, while a new investor with the same initial settings enters the market. Thus it is in the futures market in which a futures investor cannot afford one index futures contract.

## Experimental Indicators

### 3.1 Liquidity Indicators

#### 3.1.1 Effective velocity

Liquidity is a concise and effective indicator to analyze the quality and efficiency of the financial market can help determine the feasibility to clinch a deal with a reasonable price. We adopt a relative indicator called effective velocity to measure liquidity. Effective velocity is defined as turnover divided by volatility, which represents the converting speed of a unit of volatility. In order to analyze the liquidity of stock index futures, we amend the corresponding variables of effective velocity [25]. During Δt period, let *p*_1_(Δ*t*) stand for the highest transaction price, *p*_2_(Δ*t*) stand for the lowest transaction price, *h* as the tick size, *Q*(Δ*t*)/*M*(Δ*t*) as the trading volume, *M*(Δ*t*) as the holding position of the future contracts, and *VR*(Δ*t*) as the price volatility. Holding position means the value of uncovered position of the future contracts during Δt. The effective velocity *EL*(Δ*t*) is defined as:

EL(Δt)=[Q(Δt)/M(Δt)]/VR(Δt). (9)

The price volatility is defined as *VR*(Δ*t*) = [*p*_1_(Δ*t*) − *p*_2_(Δ*t*)]/*p*_2_(Δ*t*) except at the extreme condition when *p*_1_(Δ*t*) = *p*_2_(Δ*t*), here, the price volatility is represented by VR(Δ*t*) = *h*/*p*_2_(Δ*t*). The liquidity of the stock index future is an increasing function of the turnover rate, and a decreasing function of price volatility. The liquidity is high only when the turnover rate is high and the price volatility rate is low. Conversely, the liquidity is low.

#### 3.1.2 Turnover rate

Time measurement is another approach to measure market liquidity, which is based on the time required to complete the transaction. Other external and internal conditions remain unchanged—the shorter the time required to complete the transaction represents a higher liquidity. This paper imposes the turnover rate as the indicator to measure the liquidity of the market. Drawing on paper [26], which measured the turnover rate is based on the instantaneously executed commissioned orders of the total transactions. This paper imposes the daily executed orders of the total daily commissioned orders to measure the turnover rate. The specific computational process is as follows:

$$P_{buy}=\frac{{deal}_{buy}}{{Order}_{buy}}\;P_{sell}=\frac{{deal}_{sell}}{{Order}_{sell}}\;P=\frac{P_{buy}+P_{sell}}{2}.$$ (10)

Here, *deal*_*buy*_ and deal_*sell*_ represent the number of executed orders during a certain period; *Order*_*buy*_ (*Order*_*sell*_) represents the number of buy (sell) orders placed by long (short) position during a certain period; *P*_*buy*_ (*P*_*sell*_) represents the turnover rate of a long (short) position during a certain period; and *P* represents the market turnover rate during a certain period.

### 3.2 Volatility Indicator

The volatility of the financial market is an indicator of market risk since it could represent market uncertainty. Meanwhile, volatility could be a measurement of information flow. Higher volatility usually means a greater information shock to the market. Over-large volatility of the financial market means the market stability is poor. However, over-low volatility means the market reaction to information is inadequate. Paper [27] found that over-low volatility could weaken the pricing efficiency of China’s stock index futures market and impair the market liquidity, thus potentially discouraging potential investors. Thus, the volatility should be at an adequate level for the market. Paper [28] imposed high-frequency, intra-day data estimate volatility, which was a breakthrough in volatility analysis. Paper [29] found that the depicted and estimated ability of the realized volatility model based on 5-minute high frequency data was better than that of the historical volatility model based on daily return data in the Chinese stock index futures market. This paper measures the market volatility by using the standard deviation of market yield (with a 5-second interval) obtained from the experiments based on an agent-based computational method.

## The Simulation Results

In order to decrease the errors, this simulation was run five times for each experiment to observe the general law and the analyzing data are the five times mean value.

### 4.1 Liquidity Analysis

#### 4.1.1 Effective velocity

Table 3 shows the statistical results of the effective velocity. In experiment 1, the mean and median of the effective velocity are both lower. The maximum accommodating volume under the unit volatility is the largest, and the standard deviation is larger, which means the volatility is larger. From the statistical results, the existing price limit (experiment 2) is better for market stabilization than the narrower price limits (experiment 1). In experiment 5, which does not consider price limits, the mean of the effective velocity is far less than the others. Although market stability is at its best, the liquidity is poor. From the statistical results, the main problem of existing price limits is the relatively higher standard deviation, namely, that the market is not enough stable. Though the mean of effective velocity in experiment 3 is a little lower than that in experiment 2, the standard deviation is obviously lower than that in experiment 2. The simulation results in experiment 3 show that broadening the lower price limit adequately is better for market liquidity and stability. A stricter price limit or no price limit is not suitable for China’s existing stock index futures market.

**Table 3:** The Statistical Results of the Effective Velocity.

	E1.6% 6%	E2.10%, 10%	E3.10%, 20%	E4.NPL, 15%	E5. NPL, NPL
**Mean**	1236.823	1315.284	1313.811	1327.652	990.4064
**Standard Deviation**	947.0482	963.0711	769.407	673.0954	439.3404
**MAX**	4517.111	4370.124	2715.29	3329.808	1615.245
**MIN**	131.5944	235.1097	307.2347	244.301	264.8669
**median**	976.2578	1147.805	1211.435	1152.548	1003.92

#### 4.1.2 Turnover rate

Table 4 shows the statistical results of the turnover rate. In experiment 1, the mean and median of the rate are obviously lower than in the other four conditions. These results are better in the market of experiment 2, namely, the simulation market under the existing price limits. From the turnover rate indicator, the market liquidity is better under the existing price limit. Although the standard deviation for the daily turnover rate is also larger than the other four conditions, the value is in the reasonable range and is not much larger than that of the other conditions.

**Table 4:** The Statistical Results of the Turnover Rate.

	E1 6%, 6%	E2 10%, 10%	E3 10%, 20%	E4 NPL, 15%	E5 NPL, NPL
**Mean**	13.67%	20.95%	18.66%	18.44%	14.86%
**Standard Deviation**	6.04%	9.76%	6.61%	8.96%	8.75%
**Maximum**	28.79%	37.12%	30.77%	37.79%	44.87%
**Minimum**	2.07%	5.79%	6.94%	2.92%	7.11%
**Median**	13.49%	19.81%	18.88%	18.21%	14.48%

From the statistical results of the two liquidity indicators, we draw the conclusion that the existing price limit is suitable for China’s stock index futures market and that adequately broadening the lower price limits is also a good choice. Without price limits, the market liquidity would be impaired while the market volatility would increase. However, the stricter limit price is also harmful for market liquidity and could impair the price formation, which confirms the liquidity interference effect founded by previous studies.

### 4.2 Volatility Analysis

#### 4.2.1 Trend analysis of price and yield

Figures A-J in S1 File depict the trend of the average price and yield of stock index futures market under the five experiments. From the figures, the volatility clustering property of price and yield is obvious.

From Figures A-J in S1 File, one can see that the volatility clustering property of the price and the yield of stock index futures is different under different price limits, as is the trend of the stock index futures prices. This further validates the considerable impact of price limits to the stock index futures market. Figure I and Figure J in S1 File (the results of experiment 5) show the largest price and yield fluctuations of the stock index futures, which implies the relatively poor market stability under no price-limit conditions. In order to further verify the results, we conducted an ANOVA analysis.

#### 4.2.2 The ANOVA volatility analysis

As mentioned above, we obtain 21-day five-second high frequency data from each experiment run under the platform. The daily time series of yield can draw volatility descriptive statistics, thus there are 21 items of data for one experiment. This paper conducts an ANOVA analysis for each experiment in order to testify whether the market volatility has a significant difference under different price limits.

**Fig 1:**
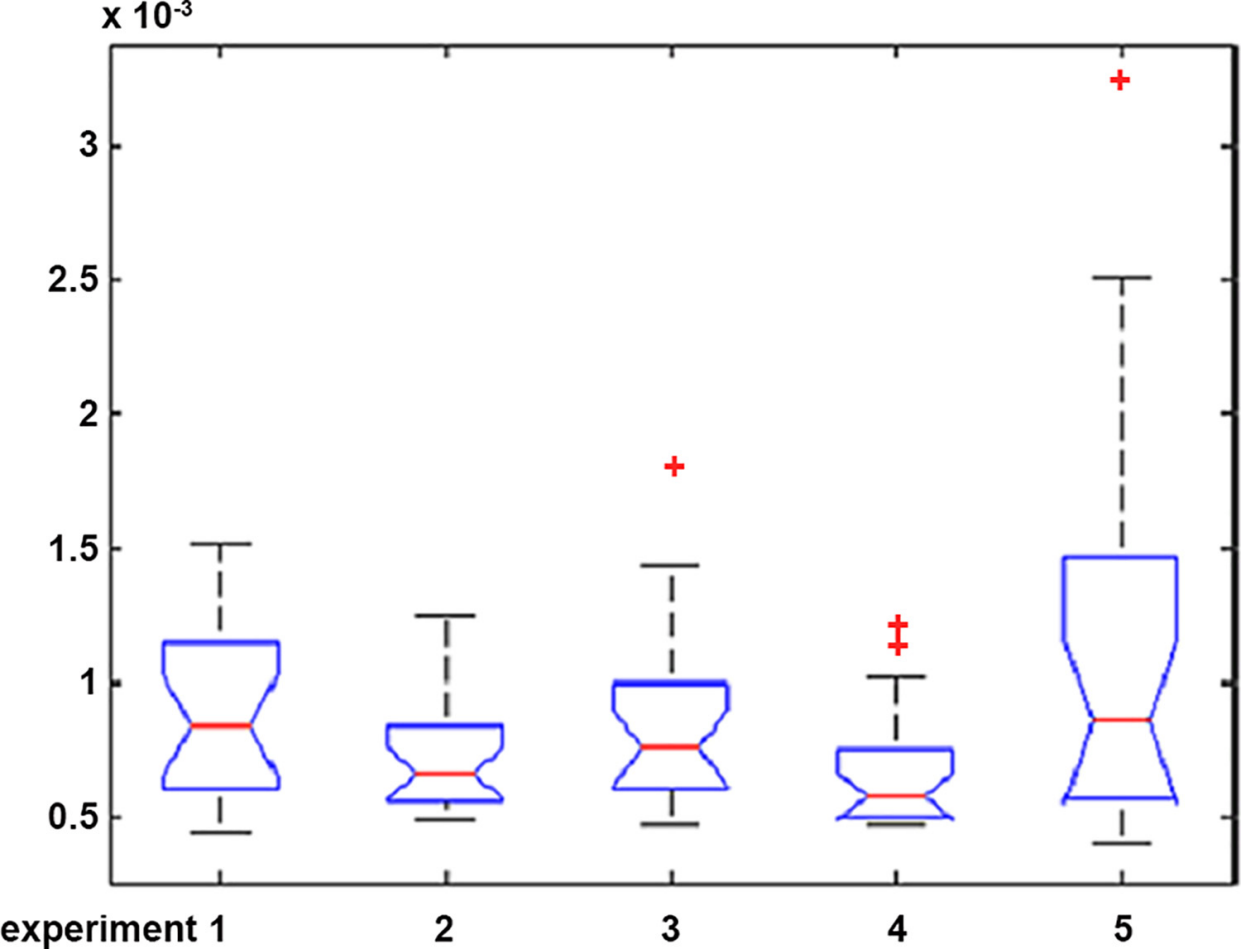
The boxplot of the stock index future’s price volatility in five experiments.

From Fig 1, it is apparent that in experiment 5, namely the simulation without setting a price limit, the median, maximum, minimum, and the height of the boxplot are larger than the other experiments. These indicators are significantly reduced in experiment 1 in which the price limit is stricter, but they are still higher than the other three experiments. From the ANOVA analysis, we draw a similar conclusion, namely that the existing price limit is in favor of market stability. In addition, adequately broadening the price limit is also good for market stability.

## Conclusion

This study focuses on the effects of price limits on the CSI 300 stock index futures market through changing the price-limit level in an artificial financial market, which is similar to the real Chinese stock market and the stock index futures market. We impose adequate liquidity and volatility indicators to compare and analyze the market stability under different conditions. The simulation results in five experiments that are quite different, which confirms the considerable impact of price limits to the stock index futures market. We find that under stricter price limits, namely up to 6% and down to 6%, the market stability is impaired due to a decrease of liquidity with an increase of volatility. The results of the experiment without price limits are worse, where liquidity is the least and volatility is at its highest of the five experiments. With the existing price limits, namely up by 10% and down by 10%, we get better results in both the liquidity and volatility aspects, though the volatility is a little higher than in other groups. However, when we broaden the lower price limit to 20%, volatility obviously decreases while the liquidity performs well. Thus, the conclusion is that maintaining the existing price limits or adequately broadening the lower price limits is favorable for market stability in the Chinese stock index futures market. A stricter price limit could impair market stability by decreasing the liquidity while increasing the volatility. Without price limits, market stability could also be impaired.

From the experience of overseas futures markets and previous studies, price limits are a low-cost technical method to control the investment risk of investors by inhibiting excessive speculations in the market and decreasing market volatility. Because the Chinese financial market is an emerging one, especially the stock index futures market, technical level risk management is better in establishing a good investment environment and enhancing investors’ confidence. From an angle of market risk management, this paper provides an important theoretical reference for research. Moreover, in future analyses, this study could be a useful extension in the consideration of adding a circuit breaker into the market to verify the influence of price limits for the Chinese stock index futures market.

## Supporting Information

S1 File Figures and Tables of data analysis. Table A: The effective velocity of the 21th experimental day’s data. Table B: The turnover rate of the 21th experimental day’s data. Figure A: The price of the stock index future in experiment 1. Figure B: The yield of the stock index future in experiment 1. Figure C: The price of the stock index future in experiment 2. Figure D: The yield of the stock index future in experiment 2. Figure E: The price of the stock index future in experiment 3. Figure F: The yield of the stock index future in experiment 3. Figure G: The price of the stock index future in experiment 4. Figure H: The yield of the stock index future in experiment 4. Figure I: The price of the stock index future in experiment 5. Figure J: The yield of the stock index future in experiment 5.
(RAR)
